# Identification of a novel *MICU1* nonsense variant causes myopathy with extrapyramidal signs in an Iranian consanguineous family

**DOI:** 10.1186/s40348-021-00116-w

**Published:** 2021-05-09

**Authors:** Fatemeh Bitarafan, Mehrnoosh Khodaeian, Elham Amjadi Sardehaei, Fatemeh Zahra Darvishi, Navid Almadani, Yalda Nilipour, Masoud Garshasbi

**Affiliations:** 1 Department of Biology, Faculty of Biological Sciences, North Tehran Branch, Islamic Azad University, Tehran, Iran; 2 Department of Medical Genetics, DeNA Laboratory, Tehran, Iran; 3 Department of Genetics, Reproductive Biomedicine Research Center, Royan Institute for Reproductive Biomedicine, ACECR, Tehran, Iran; 4 Pediatric Pathology Research Center, Research Institute for Children’s Health, Shahid Beheshti University of Medical Sciences, Tehran, Iran; 5 Department of Medical Genetics, Faculty of Medical Sciences, Tarbiat Modares University, Tehran, Iran

**Keywords:** Ca^2+^, Mitochondrial calcium uptake 1 (MICU1), Myopathy with extrapyramidal signs (MPXPS), Whole exome sequencing (WES)

## Abstract

**Background:**

Ca^2+^ as a universal second messenger regulates basic biological functions including cell cycle, cell proliferation, cell differentiation, and cell death. Lack of the protein mitochondrial calcium uptake1 (MICU1), which has been regarded as a gatekeeper of Ca ions, leads to the abnormal mitochondrial Ca^2+^ handling, excessive production of reactive oxygen species (ROS), and increased cell death. Mutations in *MICU1* gene causes a very rare neuromuscular disease, myopathy with extrapyramidal signs (MPXPS), due to primary alterations in mitochondrial calcium signaling which demonstrates the key role of mitochondrial Ca^2+^ uptake. To date, 13 variants have been reported in *MICU1* gene in 44 patients presented with the vast spectrum of symptoms.

**Case presentation:**

Here, we report a 44-year-old Iranian patient presented with learning disability, muscle weakness, easy fatigability, reduced tendon reflexes, ataxia, gait disturbance, elevated hepatic transaminases, elevated serum creatine kinase (CK), and elevated lactate dehydrogenase (LDH). We identified a novel nonsense variant c.385C>T; p.(R129*) in *MICU1* gene by whole exome sequencing (WES) and segregation analysis.

**Conclusions:**

Our finding along with previous studies provides more evidence on the clinical presentation of the disease caused by pathogenic mutations in *MICU1*. Finding more variants and expanding the spectrum of the disease increases the diagnostic rate of molecular testing in screening of this kind of diseases and in turn improves the quality of counseling for at risk couples and helps them to minimize the risks of having affected children.

**Supplementary Information:**

The online version contains supplementary material available at 10.1186/s40348-021-00116-w.

## Background

Abnormal mitochondrial Ca^2+^ handling due to biallelic *MICU1* variants causes a very rare neuronal and muscular disorder in humans termed the myopathy with extrapyramidal signs (MPXPS; OMIM #615673), characterized by impaired cognition, early muscle weakness, elevated serum creatine kinase (CK), and an extrapyramidal movement disorder [1, 2].

Mitochondrial Ca^2+^ uptake which has been long established as a key mediator of cell survival, metabolism, and death needs to be tightly regulated [3, 4]. Ca^2+^, a versatile and ubiquitous intracellular messenger [5], plays a central role in a remarkably wide range of cellular processes especially in nervous system and muscle. Calcium ions have been implicated to mediate neuronal gene expression, neuronal development and plasticity, synaptic transmission, neurotransmitter release, neuronal excitability, data processing, cognition, learning, and memory in the brain and excitation-contraction coupling, energy metabolism, adaptation to exercise, and sarcolemmal repair in muscles [2, 6–8].

The predominant mechanism among ion transporters capable of Ca^2+^ uptake into mitochondria is through a highly Ca^2+^-selective ion channel located in the inner membrane called the mitochondrial calcium uniporter (MCU), driven by electrochemical gradient across the inner mitochondrial membrane [9–12]. Mitochondrial Calcium Uptake 1 (MICU1), a regulatory subunit that shields mitochondria from Ca^2+^ overload, is required for uniporter-mediated Ca^2+^ uptake [13]. MICU1 has been suggested as a Ca^2+^ sensor which sets the threshold of extramitochondrial Ca^2+^ load for mitochondrial Ca^2+^ uptake [14, 15]. As a gatekeeper of MCU at low Ca^2+^ levels, MCU1 prevents channel opening and at high Ca^2+^ levels promotes MCU opening which allows rapid response of mitochondria to calcium signals generated in the cytoplasm [3, 16, 17].

MICU1 is a ~ 54-kDa protein which consists of 476 amino acids (NP_001182447). It contains two parts including a transmembrane helix (aa ~ 33–52) and a cytosolic C-terminus (aa ~ 53–476) which contains two EF-hand Ca^2+^-binding domains (EF1 and EF4) which help activating MCU [18].

Consistent with the clinical features displayed by patients, *MICU1* has been indicated to be highly expressed in normal mouse muscle and brain [1]. Dysregulation of *MICU1* in skeletal muscle fibers has been shown to result in sarcolemma, less contractile force, increased fatigue, and diminished capacity to repair damage to their cell membranes. In accordance with problems identified in patients, the experimental model studies characterized more pronounced muscle weakness, and greater loss of muscle mass in certain muscles [2]. Whole body knockout of MICU1 in the mouse also has been shown to cause a high probability of perinatal lethality and the survived mice have physical biochemical abnormalities, ataxia, and muscle weakness, recapitulating the problems observed in the human patients [19].

Here, we report a novel nonsense mutation c.385C>T; p.(R129*) in *MICU1* gene (NM_001195518), which is predicted to lead to a complete loss of function of *MICU1* in an Iranian patient with muscle weakness, learning disability, raised CK, elevated liver transaminases, and lactate dehydrogenase (LDH).

## Clinical presentation

A 44-year-old man with a neurodegenerative disorder was referred to the Department of Medical Genetics, DeNA Laboratory, Tehran, Iran, for genetic testing. His clinical symptoms were learning disability, muscle weakness, easy fatigability, reduced tendon reflexes, ataxia, extrapyramidal signs, gait disturbance, strabismus, elevated CK, elevated hepatic transaminases, and raised LDH. Learning disabilities were noticed during primary school, so he could not attend school. His height, weight, and head circumference were in normal range. He had progressive muscular symptoms first presented in his 10s and in his mid-20s he was completely non-ambulant and lost the ability to walk. His parents were first cousins and they were from north of Iran. Further genetic counseling revealed history of 2 other affected brothers in this family who died at the age of 46 and 48 years, respectively, one of them due to heart failure and the other due to progressive symptoms of the disease; however, no detailed medical records were available for them. The parents claimed that they had similar symptoms with the proband.

For more detailed evaluations laboratory tests, muscle tissue biopsy, electromyography, and nerve condition velocity (EMG/NCV) test were performed.

## Materials and methods

### Ethical consideration

This research has been conducted ethically in accordance with the World Medical Association Declaration of Helsinki; informed consent was obtained from all family members and the study was approved by the local medical ethics committee of DeNA laboratory, Tehran, Iran.

### DNA extraction

Genomic DNAs were extracted from the peripheral blood of the patient and all available family members by the High Pure PCR template preparation kit (Roche: product No. 11814770001).

### Targeted next-generation sequencing

**Fig. 1:**
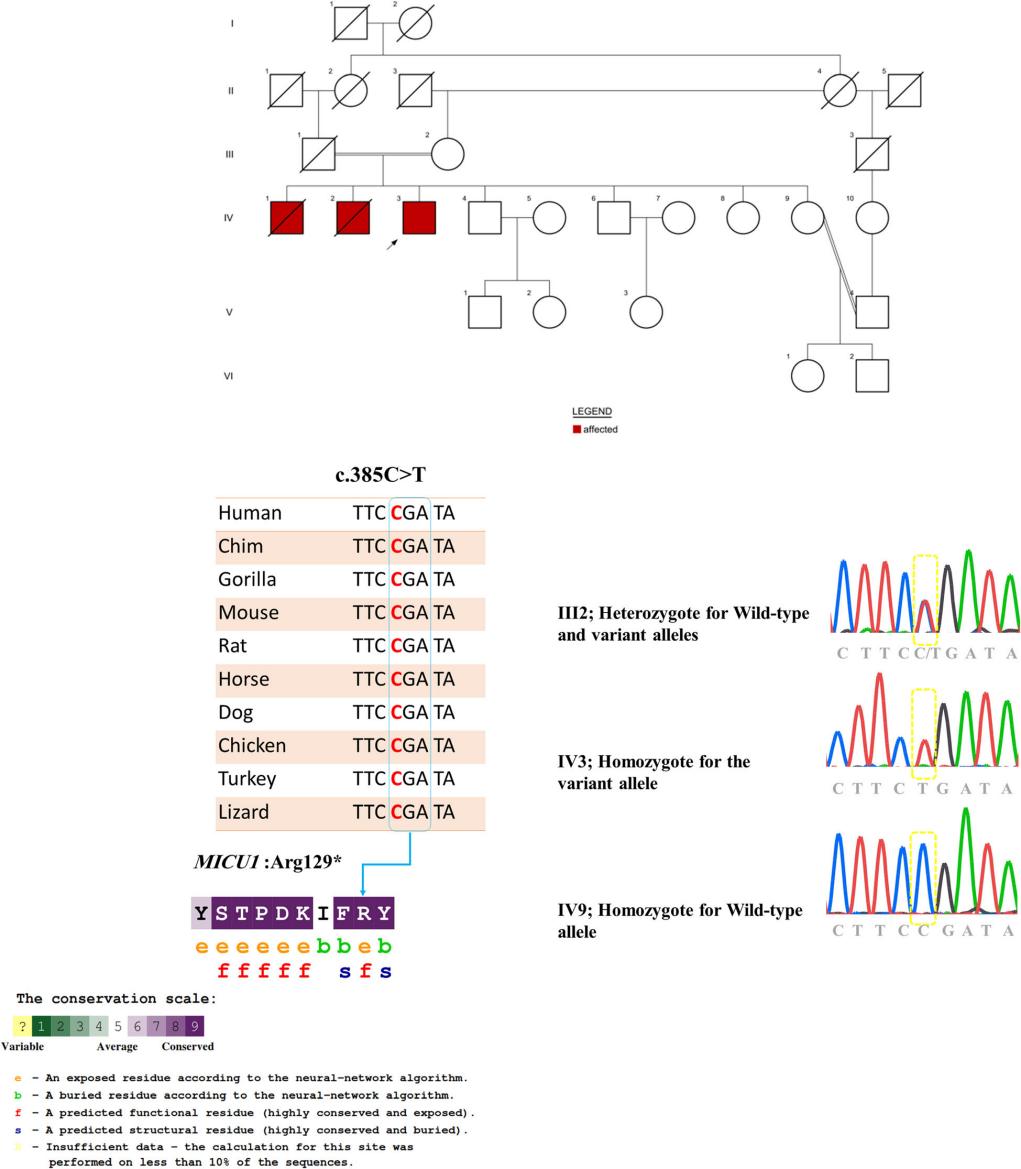
Representative pedigree, sequence chromatograms confirming the mutation, cross-species alignment, and ConSurf result of amino acid

Whole exome sequencing (WES) was performed on affected individual (IV-3; Fig. 1). Agilent’s SureSelect Human All Exon V6 kit was used to enrich approximately 60 Mb of the Human Exome from fragmented genomic DNA. The generated library was sequenced on an Illumina Hiseq 4000 platform to obtain an average coverage depth of 100. Typically, 97% of the targeted bases were covered > 10. An end to end in-house bioinformatics pipeline including base calling, alignment of reads to GRCh37/hg19 genome assembly, primary filtering of low-quality reads and probable artifacts, and subsequent annotation of variants, was applied. Reads were mapped to the reference human genome using the Burrows-Wheeler Aligner (http://bio-bwa.sourceforge.net/). Single-nucleotide variants (SNVs) and micro insertions-deletions (indels) were called using SAMtools (http://samtools.sourceforge.net/), based on filtered variants with a mapping quality score of > 20 and were annotated using ANNOVAR (http://www.openbioinformatics.org/annovar/).

Evaluation was focused on coding exons along with flanking ± 20 intronic bases. All disease-causing variants reported in Human Gene Mutation Database (HGMD) (http://www.hgmd.cf.ac.uk) and ClinVar (https://www.ncbi.nlm.nih.gov/clinvar) as well as all variants with minor allele frequency (MAF) of less than 1% in publicly available mutation and polymorphism databases such as 1000 genome project (http://www.1000genomes.org/), Exome Aggregation Consortium (ExAC) (http://exac.broadinstitute.org/), Exome Sequencing Project (ESP) (http://evs.gs.washington.edu/EVS/), and Genome Aggregation Database (gnomAD) (https://gnomad.broadinstitute.org/) were considered. We ended up with only one novel variant, c.385C>T, in *MICU1* gene. Prediction of the consequence of the c.385C>T; p.(R129*) was obtained from online databases namely SIFT (https://sift.bii.a-star.edu.sg/), and MutationTaster (http://www.mutationtaster.org/). For further consideration, the frequency of the variants was checked out on the local database, Iranome (http://www.iranome.ir/). Also, ConSurf (http://www.consurf.tau.ac.il) and UCSC database [20] was applied to check the evolutionary conservation in the region of the variant.

### Segregation analysis

Segregation analysis was investigated in the family. For this purpose, primers surrounding the region of the identified variant were designed using Primer3Plus (https://primer3plus.com/cgi-bin/dev/primer3plus.cgi) web-based server (PCR conditions and primer sequences are available upon request). Consequently, DNA sequencing of the PCR products was performed on ABI 3130 with the ABI PRISM BigDye Terminator v. 3.1 sequencing kit (Applied Biosystems, USA). Sequencing chromatograms were analyzed using Codon Code Aligner software version 8.0.2 (CodonCode Corp, USA).

## Results

### Molecular findings

The WES analysis identified a novel stop gain variant in homozygous state, c.385C>T; p.(R129*) in exon 4 of *MICU1* gene in an Iranian patient suspected to MPXPS. The homozygote normal and heterozygote state for this variant in the unaffected sister and his parents were confirmed by Sanger sequencing (Fig. 1).

**Fig. 2:**
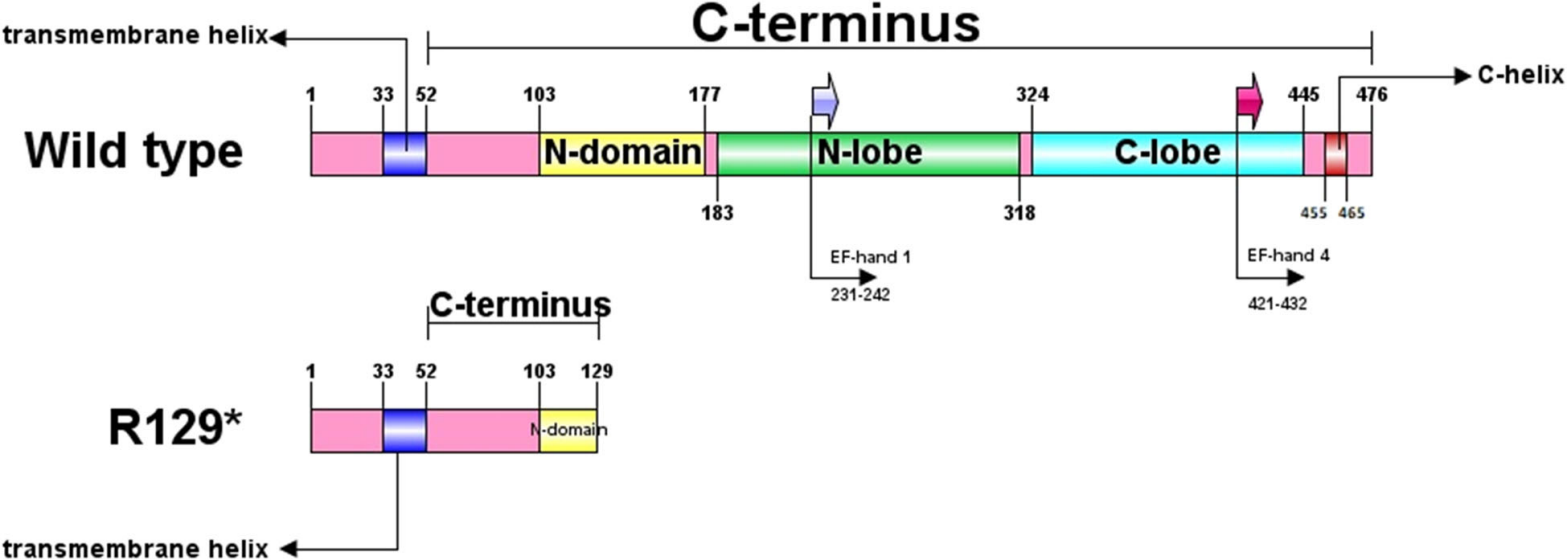
Schematic comparison of wild type and mutant predicted MICU1 structures. This nonsense mutation removed the functional chains of MICU1 protein that contribute in EF-hand structure

According to the American College of Medical Genetics (ACMG) guideline [21]: (1) Nonsense variant in *MICU1* gene, which leads to loss of function, is associated with myopathy and is a known mechanism of disease. (PVS1). (2) Pattern of inheritance is found to be autosomal recessive (PM3). (3) Co-segregation with the disease as heterozygous carriers is not affected while the homozygous individual shows myopathy phenotype. In addition, it was not found in ethnically matched healthy controls, Iranome (PS4). (4) This variant was not found in HGMD, ClinVar, 1000 genome project, ExAC, ESP, and gnomAD database (PM2). (5) Pathogenic computational verdict based on 5 pathogenic predictions from BayesDel_addAF, DANN, EIGEN, FATHMM-MKL, and MutationTaster vs no benign predictions (PP3). According to ACMG rules for combining criteria to classify sequence variants (PVS1 + PM3 + PS4 + PM2 + PP3), this variant is classified as pathogenic. The mutation p.(R129*) was also predicted to be damaging by SIFT. Cross-species alignments and ConSurf results of the variant was shown in Fig. 1. A schematic pattern of wild and truncated protein was drawn using IBS software (Fig. 2) [22].

### Laboratory tests

The patient showed raised CK up to 2081 U/L (normal, 24–195), LDH to 1352 IU/L (normal, 0–408), S.G.P.T (ALT) to 83 IU/L (normal, 0–41), and S.G.O.T (AST) to 52 IU/L (normal, 0–37).

### Muscle biopsy studies

**Fig. 3:**
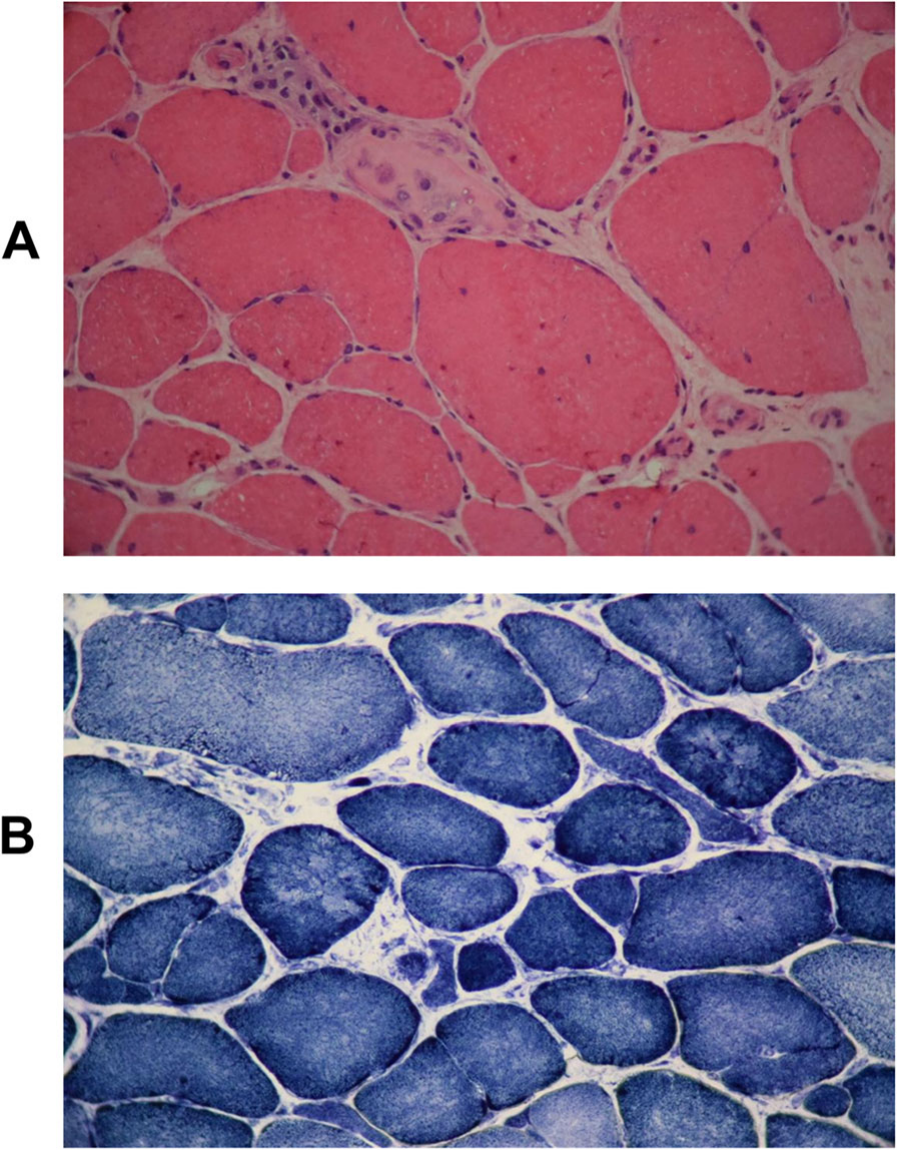
**a** Prominent fibers size variation with necrosis and myophagocytosis associated with severe endomysial fibrosis, fiber splitting, and increased internalization of nuclei (hematoxylin and eosin x400). **b** Intermyofibrillar network pattern is disrupted with presence of core-like lesions (NADH-TR x400)

Muscle biopsy from right biceps showed myopathic atrophy with dystrophic features. Multiple necrotic/regenerative fibers, myophagocytosis, and severe endomysial fibrosis were noted. Reduced nicotinamide adenine dinucleotide tetrazolium reductase (NADH-TR) staining revealed intermyofibrillar network disruption as moth-eaten fibers and core-like lesions. Adenosine triphophatase staining showed predominance of type 1 fibers and atrophy. The above histochemical pathologic findings were suggestive of muscular dystrophy, so immunohistochemical (IHC) study of dystrophin, sarcoglycans, merosin, beta-Spectrin, and dysferlin proteins was performed and sarcolemmal labeling with all the above examined antibodies was observed (Fig. 3). EMG/NCV study revealed short duration of motor unit action potential (MUAP) in two upper and lower extremities tested muscles which was in favor of myopathic changes.

## Discussion and conclusion

In this study, we report a novel biallelic *MICU1* variant, c.385C>T; p.(R129*) in an Iranian patient. Additionally, we review the literature to collect all disease-causing variants and summarize the phenotypes of all reported affected individuals. In this case, we found 44 recorded MPXPS patients in the literature including 39 patients carrying homozygous and 5 patients carrying compound heterozygous variants. Most of the homozygous patients were born to consanguineous parents. Most of the patients were from the Middle East where consanguineous marriage is ranging from 20 to 70% [23]. Up to now, 13 pathogenic *MICU1* variants have been reported in previous studies presented with the vast spectrum of symptoms even among patients carrying same pathogenic variants (Supplementary Table 1).

First, *MICU1* pathogenic variants including a homozygous splice acceptor site mutation, c.1078–1G>C, and a homozygous splice donor site, c.741+1G>A, were reported in 11 UK-Pakistani and 4 Dutch patients respectively by Logan et al. [1]. All eleven UK-Pakistani patients who carried c.1078-1G>C variant presented with developmental delay. From patients who underwent testing, all presented with elevated CK. In details, 5 patients suffered from microcephaly, 4 patients had proximal weakness, 8 patients showed extrapyramidal signs, 10 patients had learning disability, 6 patients showed speech delay, 4 patients showed skin involvement, and 3 patients had ambulation difficulties. Other features including short stature, ophthalmologic findings, and abnormal gait was observed in some cases. Muscle biopsies were available for 6 of these patients, which all exhibited myopathic features, with diffuse variation in fiber size, increased internal and central nuclei, and clustering of regenerating fibers. Necrotic fibers were rare, except in one subject. Brain MRI was available for 6 patients, out of them 1 patient had signal changes in globus pallidus, and 1 patient showed small cerebellum and 4 were normal. Four patients had skin abnormalities. All four Dutch subjects with c.741+1G>A variant presented with learning disability, ambulation difficulties, and elevated CK. Among these patients, 1 patient had short stature, 2 patients suffered from muscle weakness, 3 patients showed ophthalmologic findings, 3 patients showed extrapyramidal signs, 2 patients had abnormal gait, 2 patients showed developmental delay, 1 patient had speech delay and 2 patients exhibited skin abnormalities. Brain MRI was available for 2 patients; out of them, one patient showed linear calcification in frontal lobe and the other was normal [1].

A homozygous deletion of exon 1 of *MICU1* within a 2755-base pair deletion has been reported in 2 cousins by Lewis-Smith et al. [24]. They described a 9-year-old girl with 4 years of episodic fatigue and lethargy. She had short stature and poor growth. No neurologic and ophthalmologic signs were observed. Her cousin, a 12-year-old boy, presented with a positive Gower maneuver due to global muscle weakness, learning difficulties, developmental delay, mild hypotonia, facial dysmorphisms, long thin fingers, bilateral optic atrophy, cataracts, and pendular nystagmus. Rare atrophic fibers and increased internal nuclei showed in muscle biopsy. Echocardiography and MRI were normal. They both showed a normal blood LDH level [24].

The most common variant c.533C>T; p.(Gln185*) has been reported in 21 Middle Eastern Arab patients including 19 patients in homozygous state and 2 compound heterozygous patients concomitant with partial gene duplications. Seventeen out of 20 and 16 out of 19 cases showed elevated CK levels and liver transaminases respectively, 16 out of 20 patients presented with developmental delay, 13 out of 18 patients suffered from learning disability, 4 out of 10 had poor growth, 7 out of 16 subjects had short stature, 10 out of 19 patients showed muscle weakness, 7 out of 20 patients presented with Extrapyramidal signs, 4 out of 11 patients suffered from abnormal gait, 7 out of 11 patients characterized by ambulation difficulties, 5 out of 19 patients showed facial dysmorphisms, 10 out of 10 patients had speech delay, and 4 out of 9 patients had history of frequent falls. Lactate levels of all 10 patients tested were normal. None of 13 patients investigated for skin involvement had skin findings. Other features including seizures, calf muscle hypertrophy, ventricular septal defect (VSD), and liver involvement was observed in some cases [25–27].

A missense variant, c.386G>C; p.(R129P), was reported in two patients in compound heterozygous state accompanying by c.1A>G and c.161+1G>A variants in two studies [28, 29]. Wilton et al. [29] reported a 12-year-old female who characterized with myopathy, ataxia, abnormal gait, extrapyramidal signs, ambulation difficulties, developmental delay, learning difficulties, speech delay, generalized seizures, and multiple congenital brain malformations on MRI. She exhibited facial dysmorphisms and ophthalmologic findings. Laboratory tests indicated elevated CK levels, normal serum lactate, and normal liver transaminases [29]. O’Grady et al. [28] reported an 8-year-old boy presented with elevated CK, proximal weakness, extrapyramidal signs, learning difficulties, developmental delay, and abnormal brain MRI. Type 1 fiber predominance was diagnosed in his muscle biopsy [28].

Roos et al. [30] described a 3-year-old girl carrying a homozygous nonsense *MICU1* mutation c.553C>T; p.(Arg185*) presented with developmental delay, gait ataxia, clinodactyly, absent proprioceptive reflex, and increased CK. Muscle biopsy showed slow and fast muscle fibers affected by profound atrophy in addition to other signs of a neurogenic muscle atrophy [30]. Chérot et al. identified a compound heterozygous variant in a 4-year-old boy; c.40del; p.(Ala14Leufs*20) & c.1048C>T; p.(Gln350*), presented with intellectual disability, extrapyramidal signs, muscle weakness, dystonia, myoclonus, sensitive-motor axonal neuropathy, hypotonia, and intestinal malrotation [31].

Until now, one *MICU1* pathogenic variant, c.1295delA, has been reported [32] in Iran, a Middle East country with consanguinity rates of 38.6% of all marriages [33]. Mojbafan et al. [32] detected two affected sisters who were born to consanguineous parents. The proband was a 5-year-old girl presented with raised CK, poor weight gain, speech delay, and calf hypertrophy. She was ambulant at the age of 5 without positive Gower’s sign. Muscle biopsy showed mild myopathic atrophy with few dispersed or small groups of degenerative/regenerative fibers. Heart echocardiography revealed a mild right side enlargement and mild pericardial infusion. Electromyography and nerve condition velocity (EMG/NCV) study showed myopathic changes. She showed some extrapyramidal signs at the age of 10. Her affected sister was 2 years old who presented with speech delay and raised levels of CK, and liver transaminases. She was normal in her physical examination. EMG/NCV tests showed normal results [32].

Here, we reported the second variant, c.385C>T; p.(R129*), in a 44-year-old Iranian man with elevated hepatic transaminases, elevated CK, raised LDH, learning disability, developmental delay, easy fatigability, muscle weakness, reduced tendon reflexes, ataxia, extrapyramidal signs, gait disturbance, and strabismus. Muscle biopsy showed predominance of type 1 fibers and myopathic atrophy. Multiple necrotic/regenerative fibers, myophagocytosis and severe endomysial fibrosis, and sarcolemma were observed. EMG/NCV study revealed myopathic changes. He had 2 other similarly affected brothers who died at the age of 46 and 48 years, respectively. His extrapyramidal signs and progressive muscular symptoms first presented in his 10s and in his mid-20s he was completely non-ambulant and lost the ability to walk. These manifestations looked to be slowly progressive in line with previous studies [1, 27]. Extrapyramidal signs were observed in 5 subjects of Musa et al. study, one patient at the age of 4 years, three brothers at the mid-20s, and one patient at the age of 10 years [27]. The reported case by Mojbafan et al. also exhibited some extrapyramidal signs at the age of 10 [32]. In accordance with Musa et al. study, our patient had no skin abnormalities [27]. He also had normal height. He had no microcephaly, poor growth, and clinically observed seizures. Most of the patients who underwent testing showed normal LDH, although our case had high level of lactate in accordance with Mojbafan et al. [24, 27, 29, 32]

As mutated residue 129 had been previously reported in two cases and in our case demonstrating that R129 is a hotspot in the *MICU1* gene. The nonsense variant found in this study creates a premature protein without EF-hand motifs, which has an important role in transferring Ca^2+^ through mitochondrial membrane, and leads to a complete loss of function of MICU1 protein.

## Supplementary Information

**Additional file 1.** Previous studies presented with the vast spectrum of symptoms.

## Data Availability

The data that support the findings of this study are available on request from the corresponding author.

## Abbreviations

MICU1: Mitochondrial calcium uptake1
ROS: Reactive oxygen species
MPXPS: Myopathy with extrapyramidal signs
CK: Creatine Kinase
LDH: Lactate Dehydrogenase
WES: Whole exome sequencing
MCU: Mitochondrial calcium uniporter
EMG/NCV: Electromyography and nerve condition velocity
SNVs: Single-nucleotide variants
Indels: insertions-deletions
HGMD: Human Gene Mutation Database
MAF: minor allele frequency
ExAC: Exome Aggregation Consortium
ESP: Exome Sequencing Project
gnomAD: Genome Aggregation Database
ACMG: American College of Medical Genetics
NADH-TR: Nicotinamide adenine dinucleotide tetrazolium reductase
IHC: Immunohistochemical
MUAP: Motor unit action potential
VSD: Ventricular septal defect
