# Supplementary material for: Identification of a novel MICU1 nonsense variant causes myopathy with extrapyramidal signs in an Iranian consanguineous family
Source: Mol Cell Pediatr. 2021 May 9;8:6. doi: 10.1186/s40348-021-00116-w (PMC8107061; doi:10.1186/s40348-021-00116-w)
Supplement: Supplementary file 1 — Additional file 1. Previous studies presented with the vast spectrum of symptoms. [file 40348_2021_116_MOESM1_ESM.docx]

| **Number** | **variant(s)** | **Gender** | **Ethnicity** | **Family history** | **Age at presentation**  **Age at last follow up** | **Developmental delay** | **Speech delay** | **Intellectual and/or Learning difficulties** | **Muscular findings** | **Movement disorders**  **Extrapyramidal signs** | **Abnormal gait** | **Ambulant function** | **Short stature** | **Poor growth** | **Microcephaly** | **Dysmorphisms** | **Skin involvement** | **[Abdominal ultrasound exam](https://www.radiologyinfo.org/en/info.cfm?pg=abdominus)** | **Ophthalmological findings** | **VSD** | **Laboratory findings; Elevated Serum CK / Elevated Liver enzymes /Elevated lactate** | **Muscle biopsy** | **EMG** | **NCV(Neuropathy)** | **Brain MRI/MRS** | **Other findings** | **Ref.** |
| --- | --- | --- | --- | --- | --- | --- | --- | --- | --- | --- | --- | --- | --- | --- | --- | --- | --- | --- | --- | --- | --- | --- | --- | --- | --- | --- | --- |
| **1** | **c.1078-1 G>C**  **NM_006077** | **F** | **UK-Pakistani** | **Five affected children from a multiplex CS family**  **1; CS parents**  **2 & 3; Two affected siblings, CS parents**  **4 & 5; Two affected siblings, CS parents** | **18m**  **10yr8m** | **+** | **-** | **+** | **No proximal weakness** | **Chorea** | **-** | **Max; Runs**  **Cur; Ambulant*** | **-** | **NR** | **+** | **NR** | **Atopic dermatitis** | **NR** | **-** | **NR** | **+/ NR/NR** | **Muscle biopsies were investigated for 6 of these patients; all revealed myopathic features, with diffuse variation in fiber size,  increased frequency of internal & central nuclei & clustering of regenerating fibers without pronounced fibrosis or fatty infiltration. Necrotic fibers were rare, except in subject 11.** | **Normal** | **Normal** | **Normal** | **#** | **1** |
| **2** |  | **M** | **UK-Pakistani** |  | **22m**  **5yr** | **+** | **-** | **+** | **No proximal weakness** | **Chorea** | **-** | **Max; Climbs stairs* Cur; Runs** | **-** | **NR** | **-** | **NR** | **-** | **NR** | **-** | **NR** | **+/ NR/NR** |  | **NR** | **NR** | **NR** | **#** |  |
| **3** |  | **F** | **UK-Pakistani** |  | **18m**  **22m** | **+** | **-** | **-** | **No proximal weakness** | **-** | **-** | **Max; Ambulant**  **Cur; Climbs stairs*** | **-** | **NR** | **-** | **NR** | **-** | **NR** | **-** | **NR** | **+/ NR/NR** |  | **NR** | **NR** | **NR** | **#** |  |
| **4** |  | **M** | **UK-Pakistani** |  | **18m**  **5y10m** | **+** | **+** | **+** | **Proximal weakness** | **-** | **-** | **Max; Runs**  **Cur; Runs** | **-** | **NR** | **+** | **NR** | **-** | **NR** | **-** | **NR** | **+/ NR/NR** |  | **Normal** | **Normal** | **NR** | **#** |  |
| **5** |  | **F** | **UK-Pakistani** |  | **2yr**  **3yr2m** | **+** | **-** | **+** | **Proximal weakness** | **-** | **-** | **Max; Climbs stairs***  **Cur; Climbs stairs*** | **-** | **NR** | **-** | **NR** | **-** | **NR** | **-** | **NR** | **+/ NR/NR** |  | **NR** | **NR** | **NR** | **#** |  |
| **6** |  | **M** | **UK-Pakistani** | **NR** | **5yr**  **13yr7m** | **+** | **+** | **+** | **Proximal weakness** | **Chorea**  **Dystonia** | **-** | **Max; Runs Cur; Ambulant** | **+** | **NR** | **-** | **NR** | **-** | **NR** | **Ptosis** | **NR** | **+/ NR/NR** |  | **Normal** | **Normal** | **Signal change in globus pallidus** | **#**  **Normal respiratory chain analysis** |  |
| **7** |  | **M** | **UK-Pakistani** | **7 & 8; Two affected cousins, CS parents** | **3yr**  **13yr** | **+** | **+** | **+** | **No proximal weakness** | **Chorea** | **-** | **Max; Runs Cur; Climbs stairs*** | **-** | **NR** | **-** | **NR** | **Ichthyosis** | **NR** | **-** | **NR** | **+/ NR/NR** |  | **Normal** | **Normal** | **Normal** | **#** |  |
| **8** |  | **F** | **UK-Pakistani** |  | **3yr**  **10yr8m** | **+** | **-** | **+** | **Proximal weakness** | **Chorea**  **Dystonia** | **-** | **Max; Runs Cur; Ambulant*** | **-** | **NR** | **+** | **NR** | **Ichthyosis**  **Atopic dermatitis** | **NR** | **Optic atrophy** | **NR** | **+/ NR/NR** |  | **Myopathic changes** | **Normal** | **Normal** | **#** |  |
| **9** |  | **F** | **UK-Pakistani** | **NR** | **2yr**  **6yr** | **+** | **+** | **+** | **No proximal weakness** | **Chorea**  **Dystonia** | **-** | **Max; Runs Cur; Climbs stairs*** | **+** | **NR** | **+** | **NR** | **Ichthyosis** | **NR** | **-** | **NR** | **+/ NR/NR** |  | **NR** | **NR** | **NR** | **#** |  |
| **10** |  | **F** | **UK-Pakistani** | **10 & 11; Two affected siblings, NCS parents** | **3yr**  **27yr** | **+** | **+** | **+** | **No proximal weakness** | **Chorea**  **Paroxysmal ataxia** | **+** | **Max; Climbs stairs* Cur; Ambulant** | **+** | **NR** | **-** | **NR** | **-** | **NR** | **Nystagmus** | **NR** | **+/ NR/NR** |  | **Myopathic change** | **-** | **Normal** | **#**  **Normal respiratory chain analysis** |  |
| **11** |  | **F** | **UK-Pakistani** |  | **2yr**  **17yr** | **+** | **+** | **+** | **No proximal weakness** | **Chorea** | **-** | **Max; Runs Cur; Ambulant*** | **-** | **NR** | **+** | **NR** | **-** | **NR** | **-** | **NR** | **+/ NR/NR** |  | **Myopathic changes** | **+** | **Small cerebellum** | **#** |  |
| **12** | **c.741+1 G>A**  **NM_006077** | **M** | **Dutch** | **12 & 13; Two affected siblings, NCS parents** | **28m**  **8yr** | **-** | **-** | **+** | **Proximal weakness** | **Paroxysmal ataxia** | **+** | **Max; Runs Cur; Ambulant*** | **+** | **NR** | **-** | **NR** | **Atopic dermatitis** | **NR** | **Hypermetropia**  **Astigmatism** | **NR** | **+/ NR/NR** |  | **NR** | **NR** | **Linear calcification frontal lobe** | **#** |  |
| **13** |  | **M** | **Dutch** |  | **11m**  **37m** | **+** | **-** | **+** | **Proximal weakness** | **Paroxysmal ataxia** | **+** | **Max; Climbs stairs* Cur; Ambulant** | **-** | **NR** | **-** | **NR** | **-** | **NR** | **Hypermetropia** | **NR** | **+/ NR/NR** |  | **NR** | **NR** | **Normal** | **#** |  |
| **14** |  | **F** | **Dutch** | **14 & 15; Two affected siblings, CS parents** | **8yr**  **23yr** | **-** | **-** | **+** | **No proximal weakness** | **-** | **-** | **Max; Climbs stairs* Cur; Ambulant** | **-** | **NR** | **-** | **NR** | **-** | **NR** | **Ptosis** | **NR** | **+/ NR/NR** |  | **NR** | **NR** | **NR** | **#** |  |
| **15** |  | **F** | **Dutch** |  | **6yr**  **7yr** | **+** | **+** | **+** | **No proximal weakness** | **Dystonia** | **-** | **Max; Climbs stairs* Cur; Ambulant** | **-** | **NR** | **-** | **NR** | **Vitiligo** | **NR** | **-** | **NR** | **+/ NR/NR** |  | **NR** | **NR** | **NR** | **#** |  |
| **Number** | **variant(s)** | **Gender** | **Ethnicity** | **Family history** | **Age at presentation**  **Age at last follow up** | **Developmental delay** | **Speech delay** | **Intellectual and/or Learning difficulties** | **Muscular findings** | **Movement disorders**  **Extrapyramidal signs** | **Abnormal gait** | **Ambulant function** | **Short stature** | **Poor growth** | **Microcephaly** | **Dysmorphisms** | **Skin involvement** | **[Abdominal ultrasound exam](https://www.radiologyinfo.org/en/info.cfm?pg=abdominus)** | **Ophthalmological findings** | **VSD** | **Laboratory findings; Elevated Serum CK / Elevated Liver enzymes /Elevated lactate** | **Muscle biopsy** | **EMG** | **NCV(Neuropathy)** | **Brain MRI/MRS** | **Other findings** | **Ref.** |
| **16** | **c.386G>C p.R129P& c.1A>G**  **NM_006077** | **M** | **NR** | **NR** | **8yr** | **+** | **NR** | **+** | **Proximal weakness** | **Choreiform** | **NR** | **NR** | **NR** | **NR** | **NR** | **NR** | **NR** | **NR** | **NR** | **NR** | **+/NR/NR** | **Type 1 fiber predominance** | **NR** | **NR** | **T2 hyperintensities** | **Onset age 1yr, walked age 2.5yr** | **28** |
| **17** | **Exon 1_ 2755 bp deletion** | **F** | **NR** | **Two affected cousins**  **CS parents**  **Two other suspected individuals;**  **1. Failed to walk & died in infancy**  **2. Muscle problems** | **9yr**  **13r** | **-** | **NR** | **-** | **No muscle weakness**  **Muscle aches after 100 m of walking** | **-** | **NR** | **Ambulation difficulties due to infections or activity** | **+** | **+** | **NR** | **NR** | **NR** | **NR** | **-** | **NR** | **+/NR/-** | **NR** | **NR** | **-** | **NR** | **Normal respiratory chain analysis**  **Episodic fatigue & lethargy** | **24** |
| **18** |  | **M** | **NR** |  | **12yr** | **+** | **NR** | **+** | **Muscle weakness**  **Positive Gower maneuver**  **Hypotonia**  **Muscle aches after light exercise** | **-** | **NR** | **Ambulation difficulties due to activity** | **-** | **-** | **NR** | **Low-set big ears, a prominent chin, and long thin fingers** | **NR** | **NR** | **Bilateral optic atrophy**  **Pendular nystagmus**  **Cataracts** | **NR** | **+/NR/-** | **Rare atrophic fibers, increased internal nuclei** | **NR** | **NR** | **Normal** | **Normal respiratory chain analysis**  **Episodes of clumsiness, falls, headaches & vomiting at early childhood**  **Frequent migraines at 12 yr**  **Episodic fatigue & lethargy, poor concentration & occasional confusion** |  |
| **19** | **c.533C>T p.Q185***  **NM_006077** | **M** | **Middle Eastern Arab** | **CS parents** | **9yr**  **15yr** | **+** | **+** | **+** | **Hypotonia**  **Muscular cramps Proximal myopathy** | **-**  **No frequent falls** | **-** | **NR** | **+** | **+** | **NR** | **NR** | **-** | **No hepatomegaly** | **NR** | **-** | **+/+/-** | **NR** | **NR** | **NR** | **NR** |  | **27** |
| **20** |  | **F** | **Qatari** | **NCS parents (same tribe)** | **2yr**  **10yr** | **+** | **+** | **-** | **Hypotonia** | **+10yr**  **No frequent falls** | **+** | **Cur; Run & Climb stairs** | **+** | **+** | **NR** | **Hypertelorism, low-set ears, & hypoplastic alae nasi** | **-** | **Hepatomegaly**  **Hepatosplenomegaly** | **ptosis** | **+** | **+/+/-** | **NR** | **NR** | **NR** | **Abnormal; white matter changes** | **Tendon reflexes**  **Hemolytic anemia, low IgG, B, T, and NK cells**  **Sat unsupported at 18m Walked age 2 yr10m** |  |
| **21** |  | **F** | **Middle Eastern Arab** | **CS parents** | **10yr**  **10yr** | **+** | **+** | **+** | **No hypotonia Easy fatigability and muscle pain with walking long distances** | **-**  **No frequent falls** | **-** | **NR** | **+** | **+** | **NR** | **NR** | **-** | **Hepatomegaly**  **Increased liver periportal echogenicity** | **NR** | **-** | **+/+/-** | **NR** | **NR** | **NR** | **NR** |  |  |
| **22** |  | **F** | **Middle Eastern Arab** | **CS parents** | **11yr**  **13yr** | **+** | **+** | **+** | **No hypotonia** | **-**  **No frequent falls** | **-** | **NR** | **-** | **-** | **NR** | **NR** | **-** | **No hepatomegaly** | **NR** | **-** | **NR/NR/NR** | **NR** | **NR** | **NR** | **NR** | **Hyperactivity** |  |
| **23** |  | **M** | **Middle Eastern Arab** | **Three similarly affected brothers**  **CS parents** | **23yr**  **26yr** | **+** | **+** | **+** | **No hypotonia**  **Calf muscle, hypertrophy, Muscular cramps** | **+25, 23, 23**  **Tremors, Postural dystonia, Frequent falls** | **-** | **NR** | **NR** | **NR** | **NR** | **NR** | **-** | **NR** | **NR** | **-** | **+/+/-** | **NR** | **NR** | **NR** | **NR** | **Seizures** |  |
| **24** |  | **M** | **Middle Eastern Arab** |  | **21yr**  **24yr** |  |  |  |  |  |  |  |  |  |  |  |  |  |  |  |  |  |  |  |  |  |  |
| **25** |  | **M** | **Middle Eastern Arab** |  | **21yr**  **24yr** |  |  |  |  |  |  |  |  |  |  |  |  |  |  |  |  |  |  |  |  |  |  |
| **Number** | **variant(s)** | **Gender** | **Ethnicity** | **Family history** | **Age at presentation**  **Age at last follow up** | **Developmental delay** | **Speech delay** | **Intellectual and/or Learning difficulties** | **Muscular findings** | **Movement disorders**  **Extrapyramidal signs** | **Abnormal gait** | **Ambulant function** | **Short stature** | **Poor growth** | **Microcephaly** | **Dysmorphisms** | **Skin involvement** | **[Abdominal ultrasound exam](https://www.radiologyinfo.org/en/info.cfm?pg=abdominus)** | **Ophthalmological findings** | **VSD** | **Laboratory findings; Elevated Serum CK / Elevated Liver enzymes /Elevated lactate** | **Muscle biopsy** | **EMG** | **NCV(Neuropathy)** | **Brain MRI/MRS** | **Other findings** | **Ref.** |
| **26** | **c.533C>T p.Q185***  **NM_006077** | **F** | **Middle Eastern Arab** | **NCS parents** | **2yr**  **7yr** | **+** | **+** | **NR** | **No hypotonia** | **-** | **NR** | **NR** | **+** | **+** | **NR** | **Short neck, doughy skin**  **lax joints, down turned lower lip, tented upper lip, and mild syndactyly** | **-** | **No hepatomegaly**  **Coarse liver** | **NR** | **+** | **+/+/-** | **NR** | **NR** | **NR** | **NR** |  | **27** |
| **27** |  | **M** | **Middle Eastern Arab** | **CS parents, Two similarly affected brothers, one similarly affected sister deceased at age 2yr** | **6yr** | **+** | **NA** | **+** | **Hypotonia**  **Positive Gower sign** | **-** | **+** | **NR** | **-** | **-** | **NR** | **NR** | **-** | **NR** | **NR** | **-** | **+/+/NR** | **NR** | **NR** | **NR** | **NR** |  |  |
| **28** |  | **M** | **Middle Eastern Arab** |  | **14yr** |  |  |  |  |  |  |  |  |  |  |  |  |  |  |  |  |  |  |  |  |  |  |
| **29** |  | **M** | **Middle Eastern Arab** | **CS parent** | **10yr** | **+** | **+** | **+** | **No hypotonia** | **-**  **No frequent falls** | **-** | **NR** | **-** | **-** | **NR** | **NR** | **-** | **No hepatomegaly** | **NR** | **-** | **+/+/-** | **NR** | **NR** | **NR** | **NR** | **Seizures, Hyperactivity** |  |
| **30** |  | **M** | **Middle Eastern Arab** | **CS parents** | **3yr** | **+** | **+** | **+** | **Hypotonia** | **+4yr Choreoathetoid movement of hands and legs** | **NR** | **NR** | **+** | **-** | **NR** | **Myopathic face**  **orofacial dyskinesia** | **-** | **No hepatomegaly** | **NR** | **-** | **+/+/-** | **NR** | **NR** | **NR** | **NR** |  |  |
| **31** | **c.533C>T p.Q185***  **& Exon 9 & 10 dup**  **NM_006077** | **F** | **Middle Eastern Arab** | **CS parents** | **4yr** | **+** | **NR** | **NR** | **Hypotonia** | **-**  **Frequent falls** | **+** | **NR** | **+** | **-** | **NR** | **NR** | **-** | **NR** | **NR** | **-** | **+/+/-** | **NR** | **NR** | **NR** | **NR** |  |  |
| **32** | **c.547C>T p.Q183***  **NM_001195518** | **NA** | **Saudi Arabia** | **NR** | **NR** | **NR** | **NR** | **NR** | **NR** | **+** | **NR** | **NR** | **NR** | **NR** | **NR** | **NR** | **NR** | **NR** | **NR** | **NR** | **+/NR/NR** | **NR** | **NR** | **NR** | **NR** | **Non- specified skeletal abnormalities** | **26** |
| **33** | **c.553C>T p.R185***  **NM_001195518** | **F** | **NR** | **NR** | **3yr** | **+** | **NR** | **+** | **Muscle weakness** | **+** | **+** | **Ambulation difficulties** | **NR** | **NR** | **NR** | **Clinodactyly** | **NR** | **NR** | **NR** | **NR** | **+/NR/NR** | **Profound atrophy affecting slow and fast muscle fibres & neurogenic muscle atrophy** | **NR** | **NR** | **NR** | **Absent proprioceptive reflex** | **30** |
| **34** | **c.40del p.A14Ls*20 &**  **c.1048C>T p.Gln350***  **NM_006077** | **M** | **NR** | **NR** | **4yr** | **NR** | **NR** | **+** | **Muscle weakness** | **+** | **NR** | **NR** | **NR** | **NR** | **NR** | **NR** | **NR** | **NR** | **NR** |  | **NR/NR/NR** | **NR** | **NR** | **NR** | **NR** | **Intestinal malrotation** | **31** |
| **35** | **c.533C>T p.Q185***  **NM_006077** | **NR** | **Qatari** | **CS parents** | **NR** | **+** | **NR** | **+** | **Muscle cramps and pain** | **Abnormal movement** | **NR** | **NR** | **NR** | **NR** | **NR** | **NR** | **NR** | **NR** | **NR** | **NR** | **NR/NR/NR** | **NR** | **NR** | **NR** | **NR** | **Fatigue** | **25** |
| **Number** | **variant(s)** | **Gender** | **Ethnicity** | **Family history** | **Age at presentation**  **Age at last follow up** | **Developmental delay** | **Speech delay** | **Intellectual and/or Learning difficulties** | **Muscular findings** | **Movement disorders**  **Extrapyramidal signs** | **Abnormal gait** | **Ambulant function** | **Short stature** | **Poor growth** | **Microcephaly** | **Dysmorphisms** | **Skin involvement** | **[Abdominal ultrasound exam](https://www.radiologyinfo.org/en/info.cfm?pg=abdominus)** | **Ophthalmological findings** | **VSD** | **Laboratory findings; Elevated Serum CK / Elevated Liver enzymes /Elevated lactate** | **Muscle biopsy** | **EMG** | **NCV(Neuropathy)** | **Brain MRI/MRS** | **Other findings** | **Ref.** |
| **36** | **c.533C>T p.Q185***  **NM_006077** | **NR** | **Qatari** | **Two siblings with similar features** | **NR** | **NR** | **NR** | **+** | **Calf muscle hypertrophy** | **NR** | **NR** | **NR** | **NR** | **NR** | **NR** | **NR** | **NR** | **NR** | **NR** | **NR** | **+/+/NR** | **NR** | **NR** | **NR** | **NR** |  | **25** |
| **37** |  | **NR** | **Qatari** | **NR** | **NR** | **NR** | **NR** | **NR** | **NR** | **NR** | **NR** | **NR** | **+** | **NR** | **NR** | **Dysmorphic facial features** | **NR** | **NR** | **NR** | **NR** | **NR/NR/NR** | **NR** | **NR** | **NR** | **NR** |  |  |
| **38** |  | **NR** | **Qatari** | **NR** | **NR** | **NR** | **NR** | **NR** | **Metabolic myopathy** | **NR** | **NR** | **NR** | **NR** | **NR** | **NR** | **Mild facial dysmorphism** | **NR** | **NR** | **NR** | **+** | **+/+/NR** | **NR** | **NR** | **NR** | **NR** | **Hypoglycemia** |  |
| **39** |  | **NR** | **Qatari** | **NR** | **NR** | **+** | **NR** | **NR** | **NR** | **NR** | **NR** | **NR** | **NR** | **NR** | **NR** | **NR** | **NR** | **NR** | **NR** | **NR** | **+/+/NR** | **NR** | **NR** | **NR** | **NR** |  |  |
| **40** |  | **NR** | **Qatari** | **NR** | **NR** | **NR** | **NR** | **+** | **NR** | **NR** | **NR** | **NR** | **NR** | **NR** | **NR** | **NR** | **NR** | **NR** | **NR** | **NR** | **NR/NR/NR** | **NR** | **NR** | **NR** | **NR** |  |  |
| **41** | **c.533C>T p.Q185***  **& Partial gene duplication**  **NM_006077** | **NR** | **Qatari** | **NR** | **NR** | **+** | **NR** | **NR** | **NR** | **NR** | **NR** | **NR** | **NR** | **NR** | **NR** | **NR** | **NR** | **NR** | **NR** | **NR** | **+/+/NR** | **NR** | **NR** | **NR** | **NR** |  |  |
| **42** | **c.386G>C p.R129P**  **& c.161+1G>A**  **NM_006077** | **F** | **NR** | **NCS**  **No family history** | **12yr** | **+** | **+** | **+** | **Myopathy**  **Muscle weakness** | **+** | **+** | **Ambulation difficulties** | **-** | **-** | **-** | **Facial dysmorphism** | **NR** | **No hepatomegaly** | **Hyperopia**  **amblyopia** | **NR** | **+/-/-** | **NR** | **NR** | **-** | **White matter changes Polymicrogyria, dysmorphic basal ganglia, cerebellar dysplasia,** | **Seizures**  **Encephalopathy**  **Sat at 9m** | **29** |
| **43** | **c.1295delA**  **NM_006077** | **F** | **Iranian** | **Two affected siblings**  **CS parents** | **5yr**  **10yr** | **NR** | **+** | **NR** | **Calf muscle hypertrophy**  **No Gowers sign** | **+10yr** | **NR** | **Ambulant** | **NR** | **+** | **NR** | **NR** | **NR** | **NR** | **NR** | **NR** | **+/+/+** | **Mild myopathic atrophy with few dispersed or small groups of degenerative/regenerative fibers** | **Myopathic**  **changes** | | **NR** | **At 5yr; symptom free**  **Heart echocardiography; Mild right side enlargement and mild pericardial infusion** | **32** |
| **44** |  | **F** | **Iranian** |  | **2yr** | **NR** | **+** | **NR** | **NR** | **NR** | **NR** | **NR** |  | **NR** | **NR** | **NR** | **NR** | **NR** | **NR** | **NR** | **+/+/+** | **NR** | **Normal** | | **NR** | **Normal in physical examination** |  |
| **45** | **c.385C>T p.R129***  **NM_001195518** | **M** | **Iranian** | **Two suspected deceased brothers with similar features at 46yr and 48yr** | **44yr** | **NA** | **NR** | **+** | **Muscular dystrophy** | **+10 to 15 years** | **+** | **Ambulant difficulties at mid-20yr** | **-** | **-** | **-** | **Strabismus** | **-** | **NR** | **Strabismus** | **NR** | **+/+/+** | **Myopathic atrophy with dystrophic features**  **Multiple necrotic/regenerative fibers, myophagocytosis and severe endomysial fibrosis**  **intermyofibrillar network disruption as moth-eaten fibers and core-like lesions**  **Predominance of type 1 fibers and atrophy** | **Myopathic**  **changes** | | **NR** | **Easy fatigability, reduced tendon reflexes** | **This study** |

**CS; Consanguineous, NCS; Non Consanguineous, NR; Not Reported or not available, #; Negative for diabetes mellitus, deafness & cardiomyopathy, Max; Maximal functional achievement, Cur; Current functional level *; with support.**
